# Innate immunity and HPV: friends or foes

**DOI:** 10.6061/clinics/2018/e549s

**Published:** 2018-09-26

**Authors:** Rafaella Almeida Lima Nunes, Mirian Galliote Morale, Gabriela Ávila Fernandes Silva, Luisa Lina Villa, Lara Termini

**Affiliations:** I Centro de Investigacao Translacional em Oncologia, Instituto do Cancer do Estado de Sao Paulo (ICESP), Hospital das Clinicas HCFMUSP, Faculdade de Medicina, Universidade de Sao Paulo, Sao Paulo, BR; II Departamento de Radiologia e Oncologia, Faculdade de Medicina FMUSP, Universidade de Sao Paulo, Sao Paulo, BR

**Keywords:** Innate Immune System, Human Papillomavirus, Cervical Cancer

## Abstract

Most human papillomavirus infections are readily cleared by the host immune response. However, in some individuals, human papillomavirus can establish a persistent infection. The persistence of high-risk human papillomavirus infection is the major risk factor for cervical cancer development. These viruses have developed mechanisms to evade the host immune system, which is an important step in persistence and, ultimately, in tumor development. Several cell types, receptors, transcription factors and inflammatory mediators involved in the antiviral immune response are viral targets and contribute to tumorigenesis. These targets include antigen-presenting cells, macrophages, natural killer cells, Toll-like receptors, nuclear factor kappa B and several cytokines and chemokines, such as interleukins, interferon and tumor necrosis factor. In the present review, we address both the main innate immune response mechanisms involved in HPV infection clearance and the viral strategies that promote viral persistence and may contribute to cancer development. Finally, we discuss the possibility of exploiting this knowledge to develop effective therapeutic strategies.

## INTRODUCTION

Human papillomaviruses (HPVs) are an important group of viruses infecting the cutaneous and mucosal epithelia. HPVs cause diseases associated with high rates of morbidity and mortality, including benign lesions and cancer 1. According to the potential to cause cancer, HPV can be divided into two types: high-risk and low-risk. Low-risk types, which are mainly represented by HPV6 and HPV11, are associated with benign anogenital warts; although low-risk types are not usually associated with cancer, they may cause diseases associated with high morbidity. High-risk HPV (Hr-HPV) types, which are mainly represented by HPV16, HPV18, HPV31, HPV33, HPV35, HPV45 and other minor types, are related to cancer and precursor lesions, and Hr-HPV DNA sequences can be found in virtually all cervical tumors 2.

Cervical cancer is one of the most common cancers affecting women worldwide. According to the International Agency for Research on Cancer (IARC), the estimated number of new cases and deaths in 2012 were 527,600 and 265,700, respectively. The scenario is worse in less-developed countries, where cervical cancer represented the second most common cancer in women and was estimated to cause 230,200 of the total number of cervical cancer deaths in 2012, largely due to less-effective screening programs 3.

In addition, HPV may be associated with other anogenital tumors, although in a lower proportion. Indeed, HPV appears to be associated with 60-90% of all vaginal and anal cancers 4,5, and the virus can be found in 50% and 30-50% of vaginal and penile carcinoma cases, respectively 4,6. In addition, HPV is recognized as an etiologic cause of head and neck squamous cell carcinomas (HNSCCs); HNSCCs are mainly associated with HPV16, which is responsible for 40-80% of oropharyngeal cancers in the United States, a percentage that varies according to alcohol and tobacco use, poor oral hygiene and genetics 7,8. In general, the prognosis of HPV-positive vulvar and penile carcinomas as well as HPV-positive oral squamous cell carcinomas seems to be better than that of HPV-negative tumors 6,9.

Although most HPV infections are eliminated naturally, the persistence of Hr-HPV infection is the major risk factor for the development of high-grade cervical lesions and cervical cancer 10. To persist, HPV developed mechanisms to evade the host immune system; together with the oncogenic potential of HPV, persistence is the first step in the process leading to cancer 11.

The purpose of this review is to address the main innate immune response mechanisms involved in HPV infection clearance, HPV persistence and HPV-mediated cancer development, as well as to describe therapeutic possibilities.

### HPV Elimination and Persistence

**Figure 1:**
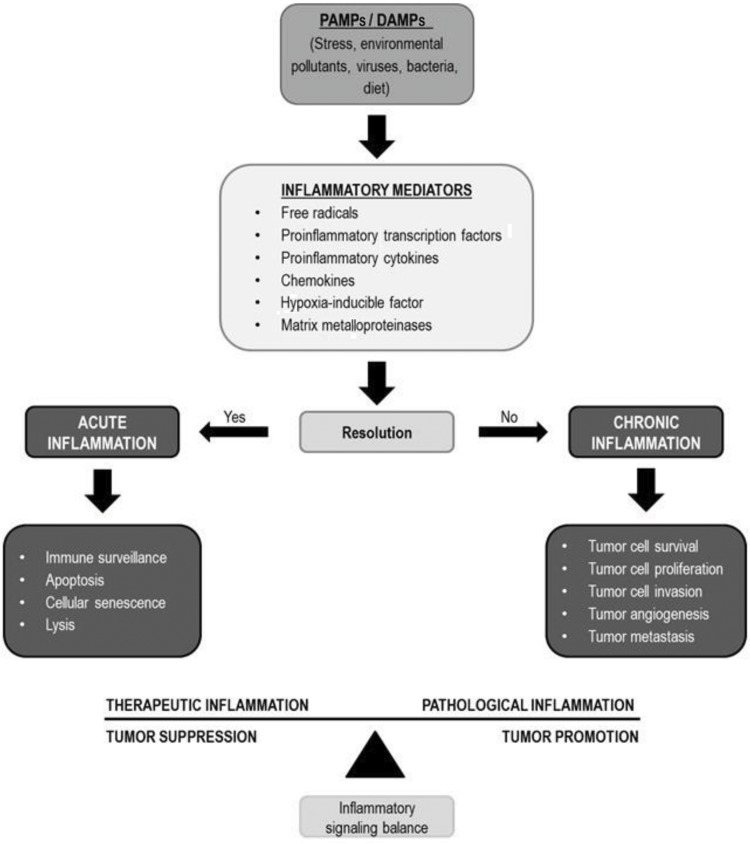
Several stimuli, including HPV infection, can trigger the release of inflammatory mediators. The balance between these mediators may favor tumor suppression or tumor promotion. PAMPs: pathogen-associated molecular patterns; DAMPs: damage-associated molecular patterns.

Several immunological factors, especially those related to innate immunity pathways, which are the first line of defense against infections, are involved in HPV recognition and elimination. The efficient triggering of the immune response is a turning point between viral clearance and persistence 12,13. As is generally observed in tumors, an inappropriate release of proinflammatory mediators and a chronic inflammatory response may contribute to cancer development (Figure 1).

In addition to innate immune cells, keratinocytes, which are both nonprofessional immune cells and targets of HPV infection, express pattern recognition receptors (PRRs). These receptors are able to identify microbial pathogens or damage signals, which are known as pathogen-associated molecular patterns (PAMPs) or damage-associated molecular patterns (DAMPs), respectively. PRRs include Toll-like receptors (TLRs), nucleotide binding oligomerization domain-like receptors (NLRs), retinoic acid-inducible gene I-like receptors (RLRs) and cytosolic DNA sensors 14,15.

Nucleic acids, which can accumulate during viral replication, are some of the microbial molecules recognized by PRRs 15. Previous studies showed that the high expression of TLR3, TLR7, TLR8 and TLR9, which recognize viral nucleic acids, is associated with HPV elimination and can be used as a predictor of clearance in HPV16-infected women. TLRs, in combination with an HPV16 E6-specific effector response, are significantly associated with viral elimination 12,13. However, it has been shown that some types of Hr-HPV are capable of compromising the innate immune response, thus not only decreasing the expression of some TLRs but also impairing important pathways involving transcription factors such as nuclear factor kappa B (NF-κB) and interferon regulatory factor 3 (IRF3), which will be discussed below, and contributing to viral immune evasion and persistence 16-18.

Polymorphisms in innate immunity genes related to HPV infection, especially infection sensors and interleukins (ILs), have been reported in the literature. Although TLR9 has been recognized as a DNA sensor, TLR9 polymorphisms do not appear to be associated with viral clearance or persistence. However, polymorphisms in other innate immunity genes, such as interleukin 1 beta (IL-1β), interleukin 18 (IL-18), NLR1 and NLR3, were shown to be associated with HPV infection and persistence 19,20.

Another sensor of foreign DNA, interferon gamma inducible protein 16 (IFI16), has been shown to be capable of controlling HPV18 replication and transcription through chromatin structure changes, thus reducing viral load and contributing to viral elimination 21.

Another antiviral mechanism involves the apolipoprotein B mRNA editing enzyme, catalytic polypeptide-like (APOBEC) protein family; members of this family are responsible for editing viral genomes, which can inhibit HPV infection. Interestingly, prolonged APOBEC activation during HPV infection can enhance genome mutagenesis, thus contributing to HPV-related cancer progression 22.

The efficiency of HPV in infection and persistence is associated with several mechanisms developed for immune response evasion. Mechanisms such as the modulation of cytokines and chemokines, downregulation of interferon (IFN) pathways, impairment of antigen presentation and reduction in the expression of adhesion molecules are directed mainly by the E6 and E7 oncoproteins 23. For example, Hr-HPV is able to inhibit cytokine production by blocking NF-κB activation upon innate immune system stimulation. Viral oncoproteins can increase the expression of interferon-related developmental regulator 1 (IFRD1), a protein responsible for recruiting histone deacetylase 1 (HDAC1) and HDAC3, thus leading to the deacetylation of NF-κB and inhibiting its ability to respond to immunological signals 17.

Additionally, to evade host innate immunity, Hr-HPV can inhibit PRR signaling through the induction of ubiquitin C-terminal hydrolase L1 (UCHL1) expression; UCHL1 blocks the activation of NF-κB and IRF3, both of which are transcription factors that induce the production of proinflammatory cytokines and chemokines 18. Additionally, Hr-HPV can downregulate the expression of inflammasome components and other downstream intermediaries of PRRs, such as genes in the IL-1β network, thus compromising not only innate immunity but also the activation of the adaptive response, which is also mediated by IL-1β 24. In addition, the direct effect of HPV16 E6 on IL-1β through its interaction with and recruitment of the E6AP ubiquitin-protein ligase, thus leading to cytokine degradation by the proteasome system, has been shown 25.

Other HPV proteins can play a role in immune escape; for example, HPV16 E2 can modulate the expression of 92 genes involved in the innate immune response, including the stimulator of interferon genes (STING), interferon kappa (IFN-κ) and interferon-stimulated genes (ISGs) 26.

The impairment of the IFN-mediated response is another resource used by HPV to evade the immune system. Viral oncoproteins reduce the secretion of IFN by keratinocytes, impair the phosphorylation of IFN pathway intermediates and compromise signaling through interferon regulatory factors (IRFs). For example, IRF1 expression is reduced in cervical tissues and cancer cell lines, and the reduced phosphorylation of IRF3 is associated with Hr-HPV infection. Thus, IFN is an important component of the innate immune response against Hr-HPV 18,27,28.

Finally, the potential for HPV to interfere with the migration and adhesion of innate immune cells and with the definition of cell phenotype must be emphasized. For example, the function of antigen-presenting cells (APCs), macrophages and natural killer (NK) cells can be compromised by HPV infection 29-32.

The abundance of dendritic cells (DCs), which are professional APCs that induce T cell-mediated immune responses, was found to be reduced in cervical intraepithelial neoplasias (CINs) 29. In addition, the expression of programmed death-ligand 1 (PD-L1), which interacts with programmed cell death protein 1 (PD-1) in order to promote T cell anergy, is higher in DCs from Hr-HPV-positive patients than in DCs from Hr-HPV-negative patients 33,34.

Thus, an inefficient innate immune response can contribute to HPV persistence, which is a well-known cause of cell transformation and tumor progression initiated by HPV infection.

### Tumor Progression: Cervical Cancer

In cervical cancer, the expression of Hr-HPV oncoproteins is essential for cell transformation, and the integration of HPV DNA into the host genome contributes to this process 35,36. These oncoproteins, especially E6 and E7, induce continuous cell proliferation and prevent apoptosis, thus favoring the accumulation of mutations in the host genome due to the inhibition of pRb and p53 function 36. However, viral oncoproteins not only affect cell cycle regulatory mechanisms but also negatively impact the innate immune response 37. Together, viral persistence and the accumulation of cellular alterations allow the development of high-grade lesions and the progression of tumors.

The immune system plays a central role in determining the outcome of HPV infection, and immune system components are important in both viral clearance and tumorigenesis 38,39; this observation is true in HPV-related cancers as well as other cancers. For example, macrophages are present in the microenvironment of solid tumors, and although they can perform antitumor functions, they may also play an important role in cancer progression. Tumor-associated macrophages (TAMs) may promote cell proliferation and angiogenesis and may restrict immune defenses 40. These different roles can be explained by the identification of two macrophage phenotypes: the M1 proinflammatory macrophage phenotype and the M2 immunomodulatory macrophage phenotype. To help in tissue repair, M2 macrophages present a profile that elicits an increased production of vascular endothelial growth factor (VEGF) and matrix metalloproteinase 9 (MMP9). However, when activated by tumors, M2 macrophages can induce basement membrane disruption, tumor growth, and metastasis 41-43.

M2 macrophages are linked to protumor responses in many ways. The antitumor properties of M1 macrophages are primarily a result of the interleukin 12 (IL-12)-dominant cytokine milieu they produce 44. When macrophages assume an M2-like phenotype, they cannot produce IL-12, which is required for the activation of the antitumor response mediated by NK cells, T helper type 1 (Th1) cells and cytotoxic T lymphocytes (CTLs). Instead, M2 macrophages produce interleukin 10 (IL-10), which induces T helper type 2 (Th2) cell polarization, thus stimulating M2 macrophage polarization in a positive feedback loop mediated through interleukin 4 (IL-4) production 44,45. Furthermore, the immunoregulatory cytokine transforming growth factor beta (TGF-β) is linked to a Th2 response, and the precursor form of TGF-β can be processed by TAMs in order to release the active molecule 46,47.

Another important mechanism contributing to tumor expansion is the recruitment of regulatory T cells (Tregs) by M2-derived C-C motif chemokine ligand 22 (CCL22). In the tumor microenvironment, Treg activity is maintained by high local levels of IL-10 but is also induced by M2 macrophages, as mentioned previously. In addition, IL-10 promotes the differentiation of naive T cells to Tregs 45,48-50. Furthermore, TAMs expressing PD-L1 can directly induce T cell apoptosis by the binding of PD-L1 to its receptor 51.

Finally, the loss of antigen-presenting capabilities in macrophages is related to the downregulation of class II major histocompatibility complex (class II MHC) expression on M2 cells 52,53. In fact, when cervical lesions progress, the number of macrophages increases, and M2 macrophages are the main macrophage population in HPV-associated tumors 30,31.

NK cells are an important part of the innate immune response against viral attack and can lyse cancer cells even without the presentation of tumor antigens 54. NK cell activation occurs through an interaction between triggering receptors, such as NKp30, NKp44, NKp46 and natural killer group 2D (NKG2D), and tumor cell ligands, which is finely balanced between inhibitory receptors and coreceptors 55. In cervical cancer, the loss of class 1 MHC expression compromises the ability of tumor cells to present viral antigens to CTLs but may make tumor cells susceptible to NK cells. In addition, CD155, which is an activating NK cell receptor, was recently reported to be upregulated in squamous cervical carcinoma 56. However, another study showed that in high-grade squamous intraepithelial lesions (HSILs) and cervical cancer associated with HPV16 infection, the expression of the NK-activating receptors NKp30, NKp45 and NKG2D (only in cervical cancer) is considerably decreased, which affects the cytolytic functionality of cells and may contribute to tumor progression 32.

Whereas acute inflammation can promote antitumor immunity in the cervix, chronic inflammation can be associated with a protumor effect, partly through the provision of growth factors for use by the tumor. In addition, the production of inflammatory nitric oxide, cyclooxygenase (COX), IL-1β and tumor necrosis factor (TNF) enhances HPV-mediated tumorigenesis 40,57-60. Thus, the resistance of Hr-HPV-infected cells to the cytostatic or cytotoxic effect of some cytokines produced in a chronic inflammatory environment could be a key step in HPV-associated tumor development.

In cervical cancer, the expression of cyclooxygenase 2 (COX-2), which is an enzyme involved in the production of proinflammatory prostaglandins, is upregulated 61, and the induction of COX-2 by HPV16 E5 through NF-κB and activator protein-1 (AP-1) has been demonstrated 62. In addition, NF-κB levels may be elevated in cervical cancer epithelial cells, and this increase is associated with poor prognosis 63. Indeed, another study has shown that the HPV16 E6 and E7 oncoproteins induce an increase in NF-κB activity 64.

Previous studies showed that despite the antiproliferative effect of TNF on primary and HPV16-immortalized keratinocytes, HPV18-immortalized keratinocytes may be resistant to this effect 65,66. Furthermore, genes associated with inflammatory responses, cell differentiation, cell death, proliferation, extracellular matrix remodeling and DNA repair were identified to be differentially expressed in HPV-immortalized keratinocytes with differential responses to the cytostatic effect of TNF 67. Taken together, these data support the idea that the acquisition of TNF resistance by HPV-infected cells may represent an important step towards malignancy.

The complete understanding of the immunological aspects of HPV infection and the cervical cancer microenvironment, including host immune components, HPV evasion and defense tactics and protumor factors, constitutes an important step in the development of new preventive and therapeutic options.

### Prophylaxis and Treatment: Vaccines

Considering the important role of the innate immune response in inflammatory processes, many studies have been carried out using receptor agonists to improve the immune response to prophylactic and therapeutic vaccines against HPV-related diseases (Table 1).

Indeed, there are three approved HPV vaccines, namely, Gardasil, Gardasil-9 and Cervarix; the first two use aluminum as the only adjuvant, and Cervarix also uses an LPS derivative that stimulates the innate immune system, thus activating TLR4 and helping to promote the death of HPV-infected cells through the activation of DCs and NK cells 68. Although Cervarix induces higher levels of neutralizing antibodies, no evidence supports Cervarix being more effective than Gardasil 69-71.

In previous studies, lipopeptides acting through TLR2 were used to stimulate the CTL response against HPV-associated tumors. Although the CTL response was useful in a prophylactic model, it could not inhibit tumor growth efficiently in a therapeutic context. The authors suggest that the depletion of immunosuppressive factors could improve the therapeutic effects of the vaccine 72. In another therapeutic vaccine model, a TLR3 agonist in a complex with an E7 peptide demonstrated a highly potent antitumor effect and induced a strong specific CTL response 73.

Interestingly, a DNA vector containing a virus-like particle sequence fused to a nononcogenic mutated E7 protein demonstrated an effect on established tumors in mice only when the vaccine was combined with TLR7 and TLR9 agonists 74. In addition, adding TLR3 and TLR7 agonists to a DNA vaccine containing the HPV16 E7 sequence promoted significant tumor regression in mice 75. Furthermore, the topical application of imiquimod (a TLR7 activator) associated with the intramuscular administration of a DNA vector containing HPV16 E7 fused to calreticulin increased the recruitment of CD8^+^ T cells to the genital tract in an orthotopic HPV16 E6/E7 syngeneic tumor model 76.

In addition, the use of flagellin as an adjuvant in order to induce a strong specific immune response through TLR5 activation has been studied as an alternative. When mixed with an intranasally administered antitumor vaccine, flagellin induced a cytotoxic response and conferred protection to mice challenged with HPV-transformed cells 77.

Additionally, the high mobility group box 1 (HMGB1) protein is related to innate immunity; HMGB1 activates TLR2/4 or the receptor for advanced glycation end products (RAGE) and can promote T cell activation. Moreover, an HMGB1 peptide displayed adjuvant properties in a mouse model when administered in combination with an E7 antigen. The data from the prophylactic and therapeutic assays indicated a substantial protective effect and showed the activation of the Th1 cellular immune response and the release of IFN-γ 78.

Apart from the agonists used in prophylactic vaccines, no other vaccines are currently approved for clinical use in the treatment of HPV-positive tumors. Nevertheless, the potential of therapeutic vaccines must be investigated since an efficient therapeutic option will not be established soon.

Persistent HPV infection is associated with the modulation of immune cells, receptors, transcription factors, cytokines, chemokines and other immune mediators, all of which play a crucial role in inducing an effective immune response against HPV. Moreover, HPV has developed several mechanisms to evade or downregulate the innate immune response, including the modulation of the PRR response, the inhibition of antiviral molecules and the inhibition of the transcription of genes associated with the immune response. In addition, during HPV-associated tumor development, innate immune cells can contribute to the establishment and progression of such tumors. Because of the important role of the innate immune response in inflammatory processes and tumorigenesis, investing in studies that target this system to improve prophylaxis against HPV infection and to elicit efficient therapeutic responses in HPV-related tumors remains relevant.

## AUTHOR CONTRIBUTIONS

Nunes RA contributed to the abstract, the introduction, “HPV Elimination and Persistence”, “Tumor Progression: Cervical Cancer” and the conclusion and revised and corrected all text and references. Silva GA contributed to “HPV Elimination and Persistence” and “Tumor Progression: Cervical Cancer”. Morale MG contributed to “Prophylaxis and Treatment: Vaccines” and to organizing the references. Villa LL prepared, revised and corrected all text. Termini L prepared, revised and corrected all text.

## Figures and Tables

**Table 1:** Potential prophylactic and therapeutic vaccines against HPV-related diseases.

Receptor	Agonist	HPV Antigen	Reference
**Prophylactic Assays**			
TLR2	Lipopeptide	E7 CTL epitope	(72)
TLR4	AS04, LPS derivative	VLP (L1)	(68)
TLR5	Flagellin	E6/E7 peptide	(77)
**Therapeutic Assays**			
TLR3	Poly (I:C)	E7 peptide	(73)
TLR3/TLR7	Resiquimod/Poly (I:C)	E7 DNA	(75)
TLR7	Imiquimod	E7-calreticulin DNA	(76)
TLR7/TLR9	Imiquimod/CpG	E7 DNA	(74)
**Prophylactic and Therapeutic Assays**			
TLR2/TLR4	HMGB1 peptide	E7 DNA	(78)
